# The wound response in fresh-cut lettuce involves programmed cell death events

**DOI:** 10.1007/s00709-018-1228-y

**Published:** 2018-02-22

**Authors:** Elena T. Iakimova, Ernst J. Woltering

**Affiliations:** 1 Horticulture and Product Physiology Group, Wageningen University, Droevendaalsesteeg 1, P.O. Box 630, 6700AP Wageningen, The Netherlands; 2 Institute of Ornamental Plants, 1222 Negovan, Sofia Bulgaria; 3 Wageningen Food and Biobased Research, Bornse weilanden 9, P.O. Box 17, 6700AA Wageningen, The Netherlands

**Keywords:** *Lactuca sativa* L., Wounding, Senescence, Cell death, Hydrogen peroxide

## Abstract

In this work, the involvement of programmed cell death (PCD) in the wound-induced postharvest browning disorder and senescence in butterhead lettuce (*Lactuca sativa* L.) fresh-cuts was studied. At the wounded (cut, bruised) sites, rapid browning, loss of chlorophyll and massive cell death, accompanied with accumulation of reactive oxygen species and increased electrolyte leakage occurred in a narrow strip of tissue adjacent the injury. The dead cell morphology (protoplast and nuclei shrinkage) together with the biochemical and physiological changes resembled necrotic PCD type. With a slight delay post-wounding, senescence associated with similar cell death features was initiated in distant non-wounded sites. In addition to necrotic PCD, both in wounded and senescing tissue, the appearance of empty cell corpses was observed, indicating that part of the cells might undergo vacuolar PCD (self-digestion of cellular content after vacuole collapse). The wounding-induced local cell death at the primary site of damage suggested that PCD may serve as a mechanism to seal-off the wound by building a physical barrier of dead cells. However, the cell death at sites remote from the wound suggests the distribution of long-distance senescence-inducing wound messengers. Trichomes in unwounded tissue often were the first to show H_2_O_2_ accumulation and dead cells; thereafter, the elevated H_2_O_2_ and cell death appeared in connecting cells and senescence progressed over larger areas. This suggests that trichomes may contribute to mediating the wound signalling leading to subsequent senescence. Our findings demonstrate that PCD is an integral part of the wound syndrome in fresh-cut lettuce.

## Introduction

The shelf life of fresh-cut lettuce (a demanded ‘ready to use’ vegetable product) is largely dependent on factors such as genetic background, developmental stage at harvest of the starting material and postharvest handling conditions (Bolin et al. 1997; Gil et al. 2012; Martínez-Sánchez et al. 2012; Witkowska and Woltering 2013, 2014; Pareek 2016). During processing, the fresh-cuts suffer from wound stress resulting from cutting, bruising, folding, pressing and other mechanical interventions that disrupt the integrity and physiological functioning of the leaf tissues. Major deterioration in the leafy fresh-cuts is pinking and browning at the wounded sites (Couture et al. 1993; Castañer et al. 1996; Cantwell and Suslow 2002; Hodges and Toivonen 2008; Pedreschi and Lurie 2015). Among others, treatments with gaseous compounds (e.g. nitric oxide (NO), ozone, hydrogen sulphide), soluble substances with antioxidant properties, chlorine and calcium-based solutions, hot water, UV radiation, high pressure, modulations of light quality and photoperiod and, genetic manipulations are shown to suppress the wound-induced browning, delay senescence, stimulate the expression of defence genes or downregulate stress- and senescence-associated genes (Coupe et al. 2003; Rico et al. 2006; Eason et al. 2014; Li et al. 2014; Mahajan et al. 2014; Iakimova and Woltering 2015; Woltering and Seifu 2015). Storage under modified (MA) or controlled atmosphere (CA) with low O_2_ (< 3%) and increased CO_2_ levels (up to 10–15%) is another technology for preventing the occurrence of browning syndrome and premature senescence. (Ballantyne et al. 1988; López-Gálvez et al. 1996a; Fonseca et al. 2002).

Although the physiological, biochemical and molecular processes involved in browning and senescence disorders have gotten appropriate attention (e.g. Hodges and Toivonen 2008; Pareek 2016), still little is known about the cellular changes underlying the wound response in fresh-cuts and particularly at the primary site of injury. Wound-induced browning is generally attributed to the production of phenolic compounds linked to the activity of polyphenol oxidase, phenylalanine ammonia lyase and peroxidase and is defined as enzymatic browning (Couture et al. 1993; Pereyra et al. 2005; López-Gálvez et al. 1996b; Degl'Innocenti et al. 2007; Saltveit and Choi 2007). Recent works suggested that lysophospholipids are the most probable primary wound signals involved in the formation of browning substances (García et al. 2017; Saltveit 2018).

An advanced view is that postharvest deterioration of fresh vegetables and fruits might be related to the occurrence of programmed cell death (PCD). It is observed that storage-induced disorders such as chilling injuries and low O_2_ and high CO_2_ disorders are often accompanied by death and sometimes disappearance of cells at specific locations. Fluids from dying cells may leak into the intercellular spaces causing macroscopic signs of deterioration (e.g. brown, sunken or water soaked lesions, scald and tissue dismantlement) (Cantwell and Suslow 2002; Coupe et al. 2003; Fernández-Trujillo and Martínez 2006; Saltveit and Choi 2007; Hurr et al. 2010; Woltering and Iakimova 2010; Eason et al. 2014; Iakimova and Woltering 2015; Cantre et al. 2017). The understanding of the role of PCD in postharvest disorders is, however, still in its infancy.

PCD is a highly coordinated process of cellular suicide. In eukaryotic systems, it is a part of the normal development and can operate as a survival mechanism at stressful circumstances (Pennell and Lamb 1997; Gunawardena et al. 2001; Lam 2004; Reape et al. 2008). According to the morphological classification introduced by van Doorn et al. (2011), plant PCD is defined in two major categories: vacuolar cell death and necrosis. Vacuolar cell death is featured by autophagic activity such as formation of lysosome-like lytic organelles, vacuolar growth and activation of vacuolar processing enzyme (VPE), tonoplast rupture and vacuole-mediated digestion of the cellular content leaving a virtually empty cell corpse behind (van Doorn and Woltering 2010). Hallmarks of necrotic cell death are swelling of mitochondria and changes of membrane permeability, early rupture of plasma membrane and electrolyte leakage, protoplast shrinkage and nucleus compaction. Necrotic PCD is associated also with respiratory decline, ATP depletion, diminished photosynthetic activity and oxidative stress-related processes. This type of cell death results in a largely unprocessed cell corpse. DNA degradation yielding a ladder pattern, due to enzymatic cleavage of DNA into oligonucleosomal fragments of 180 bp and multiples thereof, and activation of cell death related plant caspase-like proteases that are functional homologues of caspases (cysteinyl-aspartic proteases—the main executioners of animal apoptotic PCD) (Woltering 2010) may occur in both plant PCD categories. Forms of PCD expressing mixed phenotype are classified as cell death modalities. An example is the hypersensitive response (HR)—rapid local cell death occurring in plant-microbe interactions and aimed at suppressing the pathogen growth and restricting the infection to the primary site of microbial attack (Levine et al. 1994; Mur et al. 2008). Senescence and most cases of developmental PCD (e.g. xylogenesis) are generally thought to conform to the vacuolar cell death type. However, in senescing leaves and petals and in differentiating xylem vessels distinct and similar molecular patterns and physiological processes reminiscent of necrotic cell death have also been documented (Quirino et al. 2000; van Doorn and Woltering 2004, 2005, 2008; Lim et al. 2007; Price et al. 2008; Shibuya et al. 2016; Iakimova and Woltering 2017). We support the concept that the entire process of senescence is a PCD event in which autophagy in the early phases and the final culmination of cellular demise are tightly integrated. PCD—related gene expression, signalling pathways and the autophagic activity are initiated and cells acquire competence for undergoing cell death early in advance of the cell death execution phase (Yen and Yang 1998; Quirino et al. 2000; van Doorn and Woltering 2004; Shibuya et al. 2016; In this paper senescence is considered in this context.

The occurrence of PCD in senescing postharvest lettuce has been so far a subject of only few works. Wagstaff et al. (2007) found that the reduced shelf life of harvested baby lettuce leaves was associated with disruption of plastid membranes and the nuclear envelope, plasmolysis and electrolyte leakage. Electron microscopy disclosed the presence of cytoplasmic fragments in the vacuole and increased appearance of vesicles and microbodies in mesophyll and epidermal cells. The authors also observed disappearance of cells in the leaf tissue. These features resemble mainly the class of vacuolar cell death with some features of necrotic PCD. Features reminiscent of vacuolar PCD were also described in fresh-cuts of asparagus lettuce which were subjected to high pressure (above 100 MPa) processing. The excessive pressure caused cell death characterised by formation of vesicles in the cytoplasm, disappearance of chloroplasts and vacuole rupture (Zhang et al. 2015). Investigations on broccoli florets showed that quality decline during postharvest period was accompanied by cell death expressing common hallmarks of necrotic and vacuolar PCD such as DNA laddering and increasing number of TUNEL (terminal deoxynucleotidyl transferase-mediated dUTP nick end labelling) positive nuclei (a marker of double-strand DNA brakes). Tissue deterioration involved also changes in expression of the PCD-related genes *LSD1* (lesion simulating disease), Bax inhibitor (*BI*) and serine palmitoyltransferase (*SPT*), an enzyme in the sphingolipid signalling pathway (Coupe et al. 2004). Downregulation of cell death, senescence and stress-associated genes encoding for cysteine proteases such as BoCP1, BoCP2, BoCP3 and BoCP4 was documented to reduce the dehydration and delay senescence of postharvest broccoli (Coupe et al. 2003, 2004; Gapper et al. 2005; Eason et al. 2014). Bioengineering manipulation of the hormonal status is another approach for regulating the occurrence of cell death and senescence postharvest. For example, McCabe et al. (2001) reported that senescence in harvested mature heads of homozygous transgenic lettuce transformed with *Arabidopsis ipt* gene (encoding for isopentenyl phosphotransferase—enzyme from cytokinin biosynthesis and under control of the senescence-specific SAG12 promoter) was significantly retarded. Leaf senescence was also largely prevented in mutant lettuce and broccoli with suppressed ethylene synthesis genes (Henzi et al. 2000; Buchanan-Wollaston et al. 2003; Gapper et al. 2005).

Together, the mentioned findings indicate that in the leafy vegetables PCD processes may be responsible for at least some of the postharvest disorders. However, the cellular bases of wound-induced deterioration need to be better elucidated.

The rapid occurrence of browning in fresh-cut lettuce, especially at the primary site of wounding, makes this product an appropriate model to study the contribution of PCD to the wound response. In the present study wound-induced PCD events in lettuce (*Lactuca sativa* L.) fresh-cuts are addressed. Morphological, physiological and biochemical determinants of cell death were identified by applying a combined analytical approach involving microscopy, histochemical and quantitative image analyses, biochemical assay and visual observations. It is shown that PCD is an integral part of the browning and senescence syndrome in lettuce fresh-cuts. The process may serve for building a physical barrier for preventing the spread of cell death from the wounded site. The observations suggest that a wound signal generated at the primary site of injury may be communicated toward unwounded remote cells. A possible role of trichomes in mediating long-distance wound signalling leading to consecutive senescence/cell death is discussed.

## Materials and methods

### Plant material and wounding treatments

Greenhouse grown butterhead lettuce (*Lactuca sativa L.)*, cv. Cosmopolia was harvested at commercial maturity (4-week-old heads), transported to the laboratory, plastic covered and stored for 12 h in a cold room (4 °C and 96% relative humidity). The outer leaves of the heads were discarded; the leaves from the second and third whorls (mature leaves) inward from discarded ones were detached and midribs and major veins removed. With a sharp stainless steel knife these leaves were cut into pieces of approximate size 8 × 2 cm. To assess the effect of wounding, in addition to the damage at the cutting edge, in some leaf pieces, extra wounding was done by removal of a small tissue disc (0.5 cm diameter) using a cork borer, and the tissue at sites distant from the cut edge and limited to an area of approximately 1–5 mm was bruised with the tip of plastic syringe (without a needle). The additionally injured fresh-cuts were determined as group 1, (‘bruised shreds’) and the fresh-cuts subjected only to wounding at the cut surface were group 2 (‘non-bruised shreds’).

### Storage conditions

The samples were placed in plastic boxes, the bottom of which was lined with moist filter paper (Whatman grade No. 3) for preventing the desiccation and with a layer of plasticized wire mesh to avoid the contact of plant tissues with the moist paper. The boxes were covered with transparent plastic lids punctured at 16 points (approximately 1 mm diameter) to allow sufficient gas exchange with the environment and prevent accumulation of CO_2_, ethylene and other gasses released from the plant material. The samples were stored in a climate room, at 4 °C, in darkness. The experiments were undertaken with 3–4 boxes (replicates) for analyses at each time point. In total, 5 independent experiments were performed.

### Visual evaluation of deterioration and shelf life

Wound-induced browning deterioration was visually estimated by severity of browning at the cut surface and at locally wounded tissue in the ‘bruised shreds’, according to a scale previously described by Iakimova and Woltering (2015): 5—none; 4—slight; 3—moderate; 2—severe; 1—extreme browning. Senescence and shelf life were scored in the ‘non-bruised’ samples by combining overall visual quality (OVQ) and appearance of browning using two increment scale (1–9) and intermediate levels, partially adopted from Kader et al. (1973): 9—no yellowing, leaf tissues in full turgor, excellent, essentially free of defects; 7—good, minor reduction of leaf turgor, not objectionable yellowing and other defects; 5—fair, slightly to moderately objectionable senescence appearing as reduced leaf turgor, lower limit of sale appeal; 3—poor, advanced senescence expressed as excessive loss of leaf turgor and severe yellowing, limit of saleability; 1—extremely poor, very advanced senescence associated with severe tissue yellowing, necrotic lesions, desiccation and decay, not usable. The shelf life was considered terminated at OVQ rank below 5 and browning rank below 3.

### Microscopy

Microscopy was performed on leaf discs (0.5 cm diameter) isolated with a cork borer from wounded sites (cut edges and bruising sites) and from non-wounded tissue. Presented micrographs of the histological analyses are representative examples of about 75 observed microscopy fields (5 fields per micrograph) in commonly 15 micrographs collected at each time point in 3 independent experiments.

#### Histochemical detection and quantification of H_2_O_2_

Hydrogen peroxide was distinguished by 3,3′-Diaminobenzidine (DAB) staining following the protocol of Thordal-Christensen et al. (1997) and as described by Iakimova and Woltering (2015). The samples were observed and imaged under light microscope Leitz Aristoplan equipped with Nikon Digital camera DMX 1200. In the presence of H_2_O_2_ DAB is polymerised giving a visible brown stain with intensity corresponding to the amount of H_2_O_2_.

The amount of H_2_O_2_ was quantified by pixel intensity of the brown DAB deposits measured with computer application ImageJ (Image Processing and Analysis Application in Java, National Institute of Health (NIH), USA) as described in Iakimova and Woltering (2015). Pixel intensities of DAB images (in gray values, background subtracted) range from 0 to 255. Value 0 corresponds to the darkest colour and 255 to the lightest colour in the image. Higher intensity corresponds to lower H_2_O_2_ amount.

#### Histochemical detection of overall ROS

The production of overall ROS was analysed by using the fluorescent probe 2′,7′-dichlorofluorescein diacetate (DCF-DA), according to Sakamoto et al. (2005). This dye is non-fluorescent in reduced form and readily permeates the plasma membrane. Once in the cell, non-specific esterases cleave its acetate groups and the dye becomes membrane impermeable, trapped inside the cell. When oxidised by H_2_O_2_, hydroxyl, peroxyl and other free oxygen radical products, DCF-DA is converted to the green fluorescing form 2′,7′-dichlorofluorescin and ROS appeared in green. Overall ROS were visualised in leaf discs (0.5 cm diameter), collected as described above. The samples were washed with distilled water and incubated in presence of 10 μmol l^−1^ DCF-DA for 60 min at room temperature, in darkness. The fluorescence emitted from stained ROS was detected under Zeiss Axioskop fluorescent microscope equipped with filter combination excitation/emission wavelength 490/525 nm and with Nikon Digital camera DMX 1200 for imaging.

#### Quantification of the fluorescence emitted from chlorophyll

The change in chlorophyll was estimated by the red fluorescence emitted at wavelength 490/525 (excitation/emission) by using Zeiss Axioskop fluorescent microscope equipped with Nikon Digital camera DMX 1200. Chlorophyll amount was quantitatively expressed in pixel intensity by analysing the images using ImageJ. Pixel intensity (indicating the presence of chlorophyll) was measured similarly to the described for H_2_O_2_ quantification. However, opposite to the readings for H_2_O_2_, the higher pixel intensity corresponds to higher chlorophyll amount. Zero value of grey corresponds to the lower level of fluorescence and value 255 represents the highest level.

#### Cell death determination

Cell death was analysed by Evans Blue and propidium iodide (PI) staining.

Evans Blue staining of the dead cells (the dye is excluded from the living cells) was performed according to Keogh et al. (1980), with slight modifications as described in Iakimova and Woltering (2015). The dead cells were identified by the blue coloration of their content (Evans Blue positive cells). Observations and imaging were done by light microscope Leitz Aristoplan equipped with Nikon Digital camera DMX 1200.

The dead cells were also distinguished by labelling with the fluorophore PI which penetrates the damaged plasma membrane and the nucleus. This dye emits red fluorescence after binding to DNA by intercalating between the bases with little or no sequence preference and with a stoichiometry per 4–5 base pairs of DNA. The stained cells are defined as PI positive. Following the manufacturer instructions (Molecular Probes, Inc.), leaf discs were incubated in 500 nmol l^−1^ PI (in dH_2_O_2_) for 1–5 min and then rinsed with dH_2_O_2_. The observations were done under fluorescent Zeiss Axioskop microscope, excitation/emission filters 530/625 nm. Images were taken with Nikon Digital camera DMX 1200.

### Electrolyte leakage assay

In addition to Evans Blue and PI stainings, cell death was estimated by electrolyte leakage (EL) which is a marker of the permeability of the cellular membranes. The EL was determined by measuring the electrical conductivity (EC) according to Song et al. (2012) and as earlier described by Iakimova and Woltering (2015), and expressed in percentage, calculated using the formula: EL (%) = EC1_initial conductivity_/EC2 _total conductivity_ × 100 where EC1 and EC2 are in microsiemens (μS).

### Data analysis and artworks

The data were subjected to Student’s *t* test, one-way analysis of variance (ANOVA) at probability level *P* ≤ 0.05 (IBM SPSS Statistics). Graphic artworks were done by using MS Office Excel; the images were combined by MS Office Power Point and sized by Windows 64 Bit Software IrfanView.

### Chemicals

All chemicals (if not otherwise indicated) used for the assays were purchased from Sigma-Aldrich Chemie B.V., Zwijndrecht, The Netherlands.

## Results

### Shelf life and tissue deterioration

**Fig. 1:**
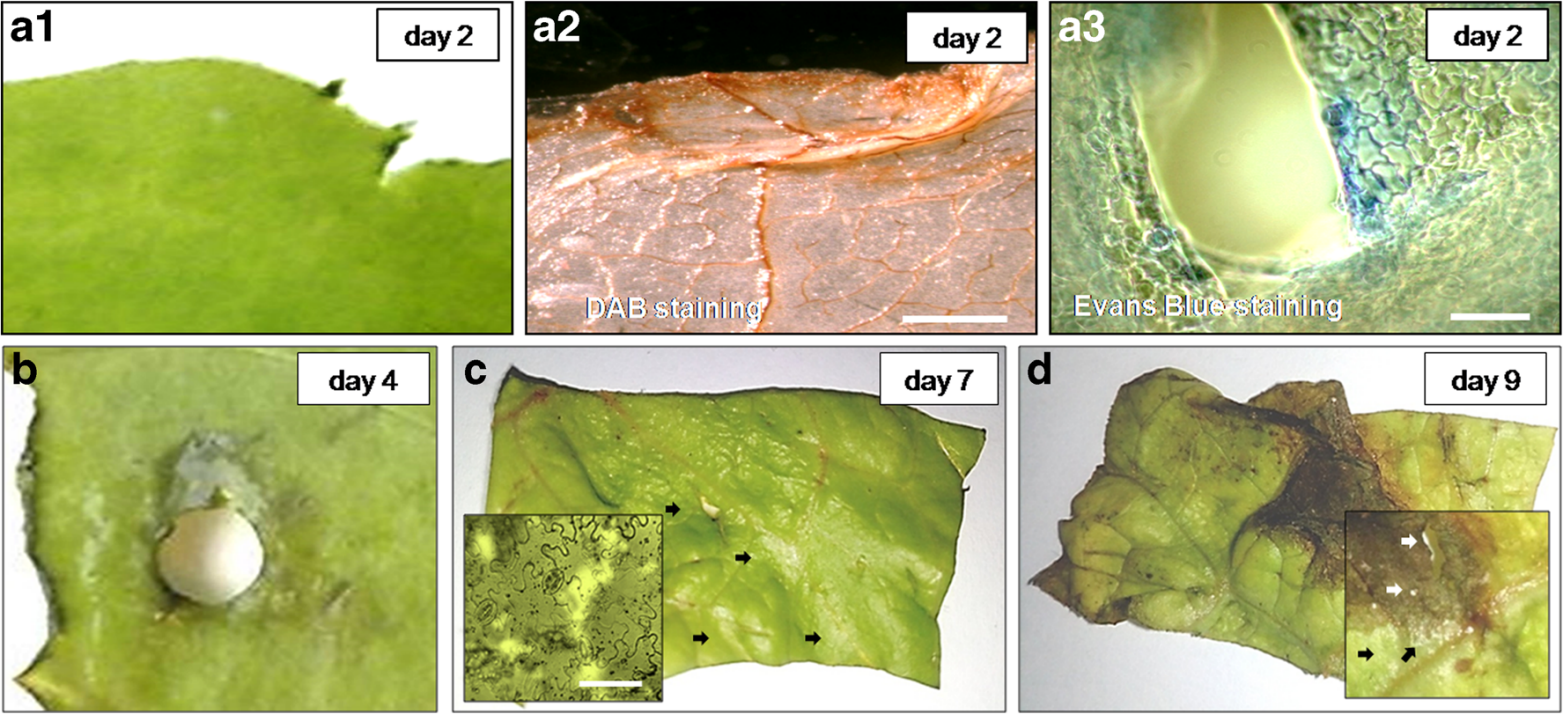
Senescence, wound-induced browning and cell death in lettuce fresh-cuts, stored at 4 °C **a**1 Slight browning at the cut edge (non-labelled tissue). **a**2 H_2_O_2_ production at the area showing initial browning, DAB staining, chlorophyll removed—the tissue appears in brown due to the presence of H_2_O_2_. **a**3 Cell death surrounding an injured site; following Evans Blue staining the dead cells appear in blue (**a**1–3), day 2. **b** Tissue browning in area adjacent a site injured with a cork borer, day 4. **c** Senescing shred on day 7; note sites in which cells have disappeared (arrows); inset shows area with vanished cells. **d** Senescing shred on day 9; visible is severe browning close to the cut edge, a large necrotic lesion of entirely necrotized tissue and disappearance of cells inside and in vicinity to it (inset, arrows). *Scale bars* = **a**2 500 μm, **a**3 50 μm, **c** (inset) 100 μm

**Fig. 2:**
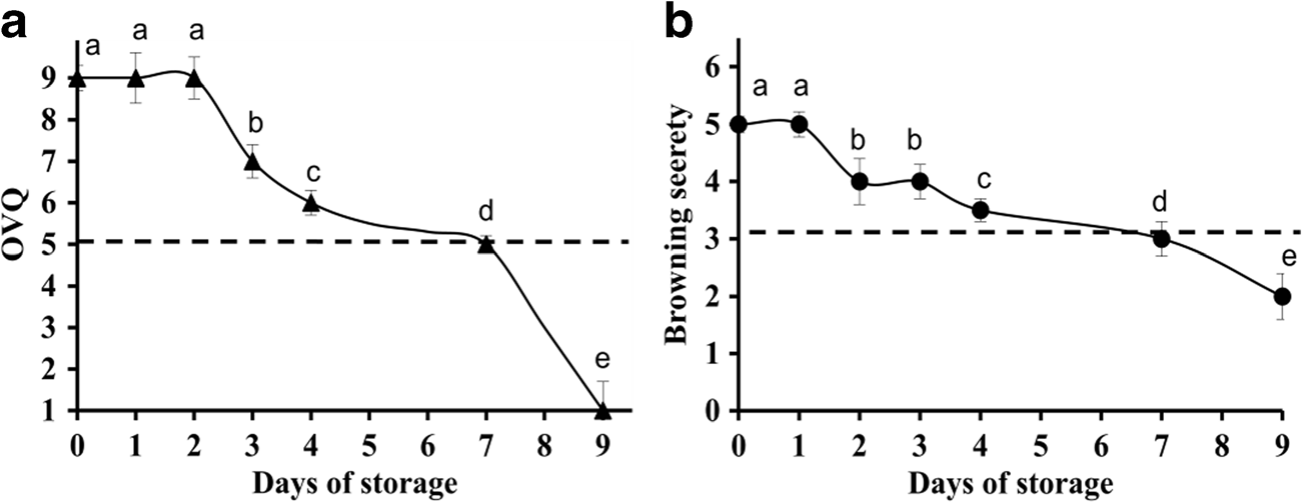
Time course and severity of deterioration of lettuce fresh-cuts stored at 4 °C **a** Overall visual quality (OVQ). **b** Browning severity. Dashed lines indicate the lower limit of consumer acceptance. Presented values are means ± SEM _(n–1)_ (*n* = 20); 4 replicates per time point of fresh-cut samples prepared from 5 lettuce heads in each of 5 independent experiments. Data indicated with same letters do not differ significantly from each other at *P* ≤ 0.05

The OVQ and the occurrence of browning in the ‘non-bruised’ fresh-cuts were visually evaluated from day 1 to day 9 of storage (Figs. 1 and 2). Yellowing and slight browning near the cut edge and around the other injured sites initially appeared on day 2 and the severity was increasing until the end of shelf life (Figs. 1 a1, b–d and 2b). The first symptoms of senescence (slight yellowing and minor loss of leaf turgor) in ‘non-bruised shreds’ were noticed around day 3 and in the following days the fresh-cuts completely senesced (Fig. 2a). After about 7 days, the quality reached the limit of acceptability (levels of OVQ below 5 and browning score below 3) (Figs. 1b and 2a, b). On day 9, necrotic lesions consisting of entirely disintegrated tissue were observed (Fig. 1d). At some places inside the necrotic lesions and also in non-necrotic senescing areas, the cells disappeared and this was already noticed on day 7 (Fig. 1c, d). In ‘bruised shreds’, rapid browning was observed at the cut edges and at the bruised sites (Fig. 1a1 and 1b) and early cell death was detected within the initially browning areas in a narrow strip of tissue surrounding the wounds (Evans Blue positive blue coloured cells) (Fig. 1a3). A symptom of PCD occurring rapidly after the wounding was also the increased level of H_2_O_2_ on day 2 (Fig. 1a2). The first areas with senescing tissue in ‘bruised shreds’ appeared distant of the wounded sites approximately 1 day after the initiation of visible browning. Further, senescence in unwounded tissue of these shreds developed in a manner similar to that in the ‘non-bruised’ shreds. Until the end of shelf life, the browning in both groups of samples remained confined to a zone bordering the cut edge and in the vicinity to the local bruises (Fig. 1b–d). These data showed that the wounding accelerates senescence in unwounded tissue and that, although with increasing severity, the browning deterioration developed only in vicinity of wounded sites.

### Chlorophyll loss

**Fig. 3:**
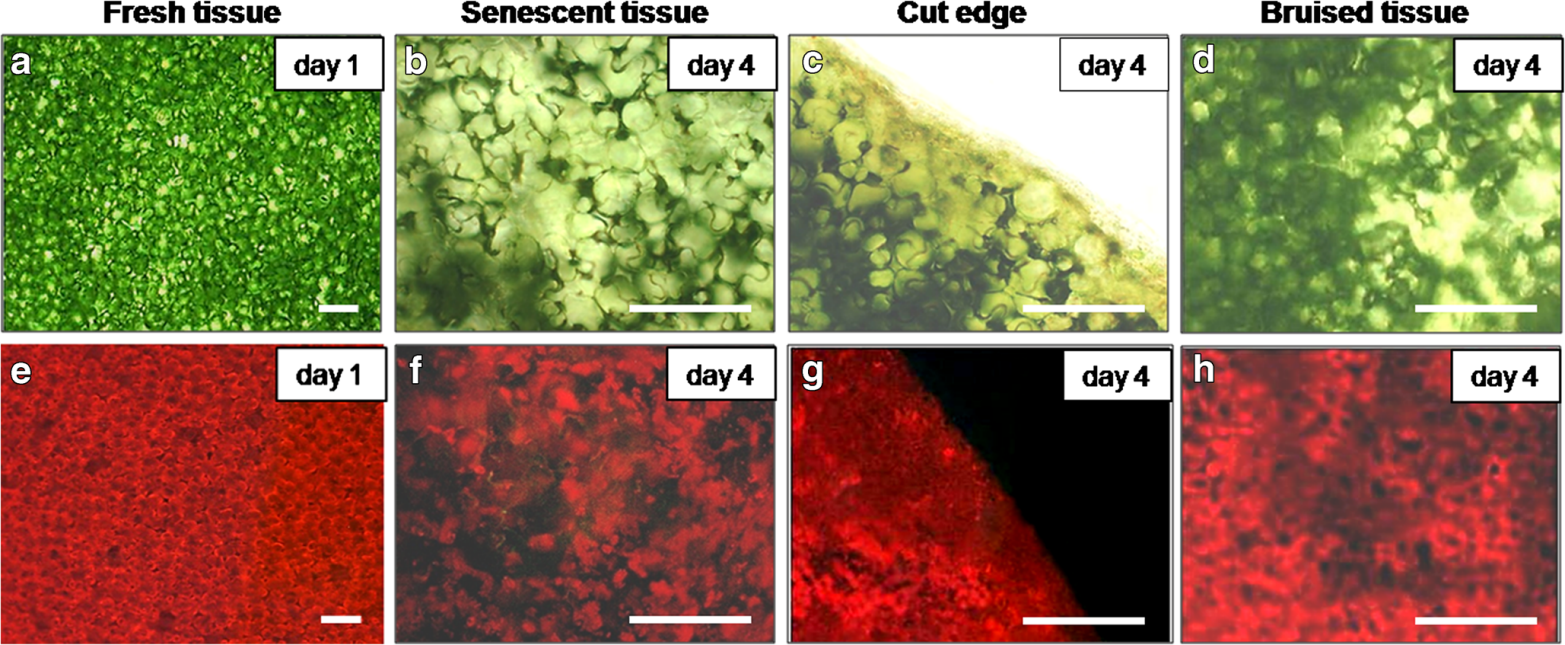
Microscopy observations on chlorophyll loss in senescing ‘non-bruised’ and ‘bruised’ shreds of lettuce stored at 4 °C **a** Day 1 of storage. **b** Senescing area, day 4. **c** Cut edge, day 4. **d** Bruised site, day 4. **e**–**h** Fluorescence of chlorophyll in panels **a**–**d**, respectively. **a**–**d** Light microscopy; **e**–**h** Fluorescent microscopy. At sites loosing chlorophyll the red fluorescence is fading or not detectable. *Scale bars* = **a** and **e** 200 μm, **b**–**d** and **f**–**h** 100 μm

**Fig. 4:**
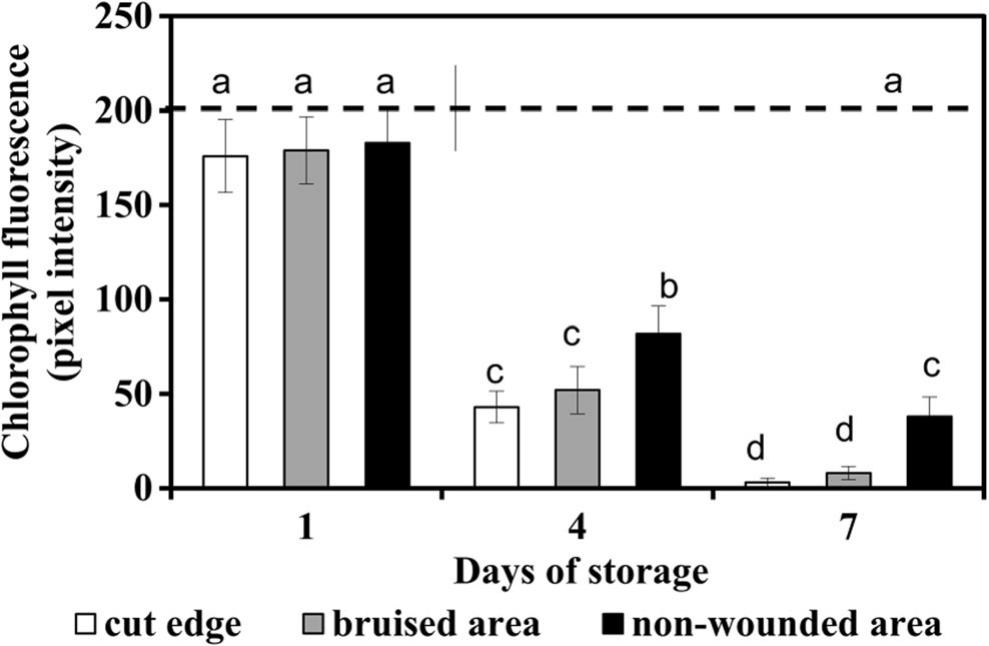
Fluorescence of chlorophyll at wounded and senescing sites of lettuce fresh-cuts stored at 4 °C Fluorescence is quantified by pixel intensity. Initial value (day 0) is shown as dotted line. Presented values are means ± SEM _(n–1)_, (*n* = 25). Quantification was done in 5 non-overlapping microscopy fields in each of at least 5 representative micrographs collected from 3 independent experiments; each separate experiment was carried out with fresh-cut samples prepared from 5 lettuce heads. Data indicated with same letters do not differ significantly from each other at *P* ≤ 0.05

Red fluorescence of chlorophyll was visible by microscopy and quantified as a decrease in pixel intensity (Figs. 3 and 4). In freshly prepared fresh-cuts and after 1 day of storage, chlorophyll loss was not yet observed (Figs. 3a, e and 4). At later time points, the fluorescence gradually declined. A decrease of chlorophyll in wounded and senescing areas was very clear on day 4 of storage (Figs. 3b–d, f–h); chlorophyll more rapidly disappeared at the cut edge and bruised sites than in the non-wounded sites (Fig. 4). In the samples taken from brown tissue surrounding, the wounded sites (cut edge or bruises), on day 7 (end of shelf life, images not shown), the fluorescence was almost completely absent whereas in non-bruised senescing tissue it remained detectable albeit with lower intensity (Fig. 4). These observations showed that chlorophyll loss initially was restricted to a small area around the wounds, whereas senescence-associated chlorophyll breakdown in non-wounded senescing tissues occurred over the whole leaf area and was delayed in comparison to wounded sites.

### Production of H_2_O_2_ and overall ROS

**Fig. 5:**
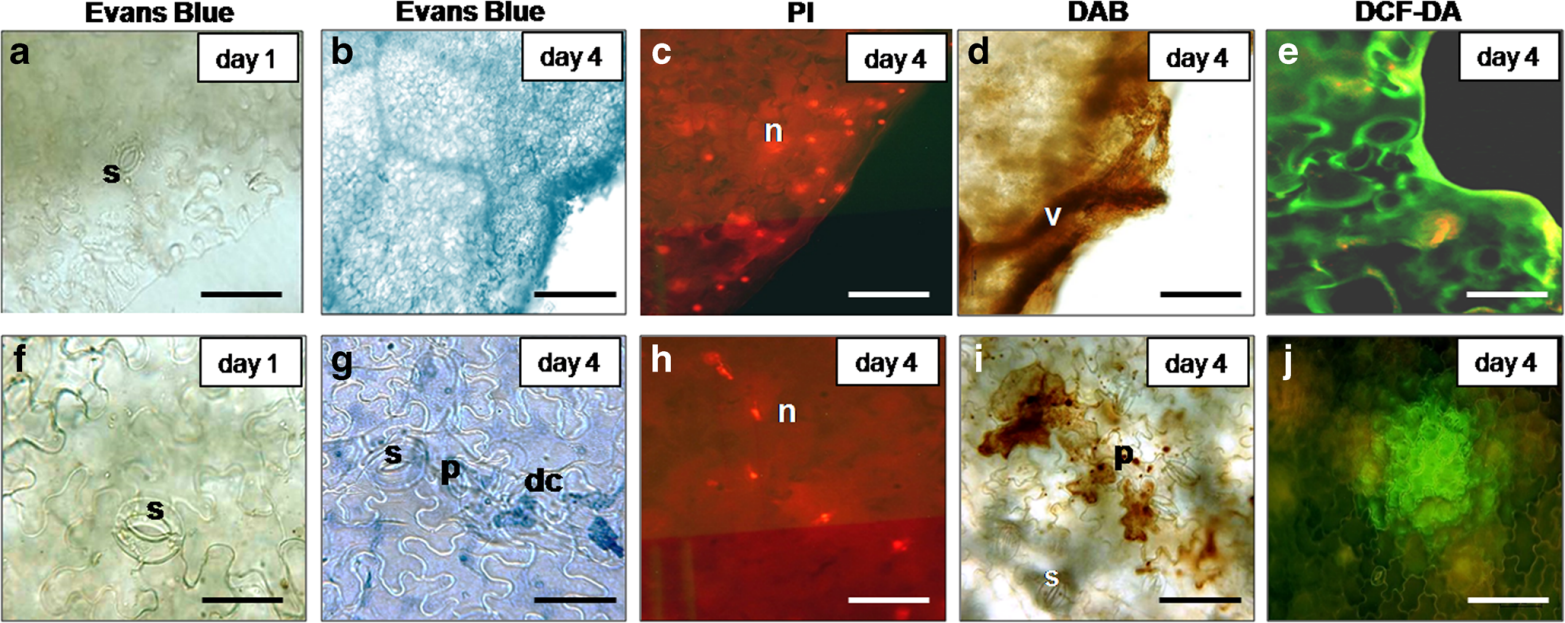
Wound-induced cell death response in lettuce fresh-cuts stored at 4 °C **a** Evans Blue stained cut edge; the lack of blue coloured tissue indicates a lack of dead cells day 1. **b** Evans Blue stained dead cells at the cut edge; note the blue stained tissue, day 4. **c** PI stained dead cells in vicinity to cut edge, day 4; note the PI positive (red fluorescing) condensed nuclei. **d** H_2_O_2_ accumulation in vicinity to cut edge, day 4; DAB staining; note the brown coloured deposits. **e** Accumulation of overall ROS in vicinity to cut edge, day 4. DCF-DA staining; note the green fluorescence. **f** Evans Blue stained bruised leaf area; no blue labeled dead cells are detected, day 1. **g** Evans Blue stained bruised area; visible are dead cells with shrunken protoplast (in blue), day 4. **h** PI stained nuclei (bright red fluorescence) in dead cells in bruised area, day 4. **i** H_2_O_2_ accumulation in cells with shrunken protoplasts in bruised area; note the brown DAB deposits, day 4. **j** ROS accumulation (green fluorescing cloud) in bruised area, day 4; DCF-DA staining. **a**, **b**, **d**, **f**, **g** and **i** Light microscopy; **c**, **e**, **h** and **j** Fluorescent microscopy. dc Dead cell, n Nucleus, p Protoplast, s Stoma, v Vessel. *Scale bars* = **a**–**e**, **i** and **j** 100 μm, **f**–**h** 50 μm

**Fig. 6:**
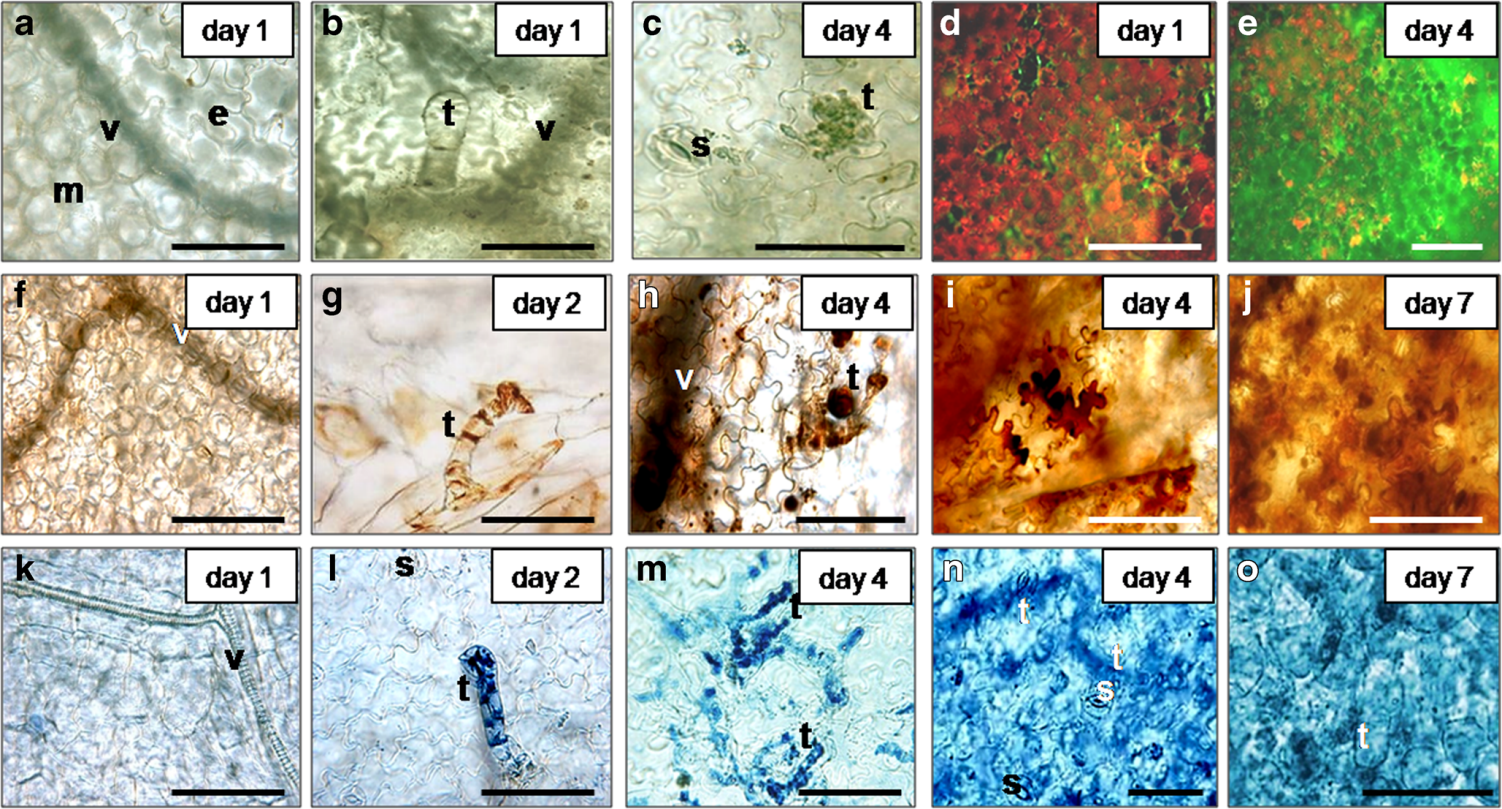
Cell death in senescing lettuce fresh-cuts stored at 4 °C **a** Non-senescing area, day 1; on the right of the vessel—part of the fresh-cut with intact epidermis; on the left - the mesophyll layer with epidermis removed. **b** Living trichome, day 1. **c** Dead trichomes, day 4. **a**–**c** Chlorophyll removed. **d** Overall ROS (green fluorescence) in non-senescing area; the red fluorescence is emitted from chlorophyll in the living cells, day 1. **e** Overall ROS in senescing area, day 4. **f** H_2_O_2_ in non-senescing area; H_2_O_2_ is detectable by the brown DAB labelling inside the vessels, day 1. **g** H_2_O_2_ in dead trichome and in epidermal cells underneath; note the brown DAB deposits, day 2. **h** H_2_O_2_ in dead trichomes; xylem vessel heavily loaded with H_2_O_2_, day 4. **i** H_2_O_2_ in senescing area, day 4. **j** H_2_O_2_ in senescing area, day 7. **i** and **j**—note the spread and increasing intensity of the brown coloration. **k** Evans Blue stained non-senescing area; blue coloured dead cells are not detectable, day 1. **l** Dead cells in trichome; note the dead Evans Blue positive (blue) cells in the upper part and the living cells (Evans Blue negative) at the base of trichome, day 2. **m** Evans Blue stained dead cells in several dead trichomes and in the connected epidermal cells, day 4. **n** Cell death in senescing area, day 4. **o** Cell death in senescing area, day 7. **a**–**c** and **f**–**o** Light microscopy; **d** and **e** Fluorescence microscopy. **d** and **e** DCF-DA staining. **f**–**j** DAB staining. **k**–**o** Evans Blue staining. e Epidermis, m Mesophyll, s Stoma, t Trichome, v Vessel. *Scale bars* = 100 μm

**Fig. 7:**
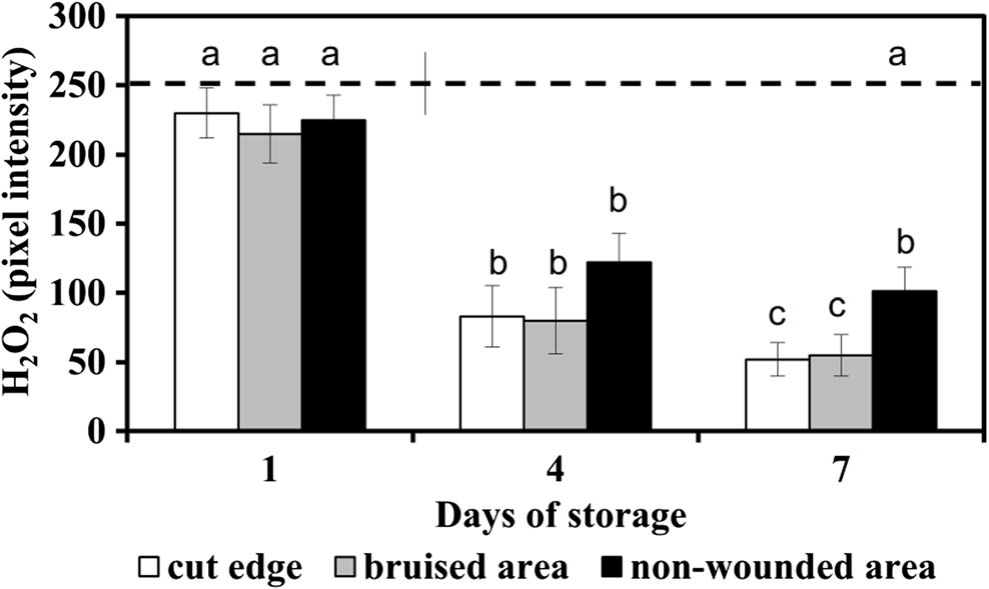
Production of H_2_O_2_ in senescing and wounded lettuce fresh-cuts stored at 4 °C The amount of H_2_O_2_ is quantified by pixel intensity of DAB deposits. Initial value (day 0) is shown as dotted line. Presented values are means ± SEM _(*n*–1)_, (*n* = 25). Quantification was done in 5 non-overlapping microscopy fields in each of at least 5 representative micrographs collected from 3 independent experiments; each separate experiment was carried out with fresh-cut samples prepared from 5 lettuce heads. Data indicated with same letters do not differ significantly from each other at *P* ≤ 0.05

The generation of ROS, including H_2_O_2_ was analysed histochemically by using specific labelling. DAB staining (formation of brown-coloured deposits) revealed massive H_2_O_2_ accumulation close to the cut edge. In bruised areas, H_2_O_2_ started to increase on day 2 (Fig. 1a2) and was well expressed after 4 days of storage (Fig. 5d, i). The diminution of pixel intensity (indicating H_2_O_2_ quantity) in the images supported this observation (Fig. 7). In comparison to day 0, no difference in H_2_O_2_ amount was detected on day 1. The level of H_2_O_2_ at the cut edge was increasing with advancement of tissue browning and cell death in vicinity of the wounds (Figs. 2b, 5b, d and 7). High levels of H_2_O_2_ were found also in xylem vessels and in their neighbouring cells within injured sites (Figs. 5d and 6h). These results pointed that enhanced H_2_O_2_ was confined to wounded areas.

Maximum production of H_2_O_2_ in senescing ‘non-bruised shreds’ was detected on day 4 and this did not change with the progression of deterioration until day 7 (Figs. 6i, j and 7). At the end of shelf life, however, H_2_O_2_ remained lower in senescing tissue than at the cut edge and at otherwise wounded tissue (Fig. 7). This suggested that with advancement of senescence at certain time point, the capacity of senescing cells to produce H_2_O_2_ might diminish.

The labelling with DCF-DA resulted in clearly distinguishable green fluorescence indicating substantial increase in the generation of overall ROS at the cut edge and in bruised sites (Fig. 5e, j). In comparison to fresh tissue at day 1 (Fig. 6d), ROS accumulation was spread over larger area by day 4 (Fig. 6e). These observations showed that enhanced generation of H_2_O_2_ and other ROS occurs close to the sites of injury and in senescing tissue. In general, the massive ROS, including H_2_O_2_, were detected in the cells most probably determined to die (Figs. 5b–d, g–i and 6i, j, n, o).

### Electrolyte leakage

**Fig. 8:**
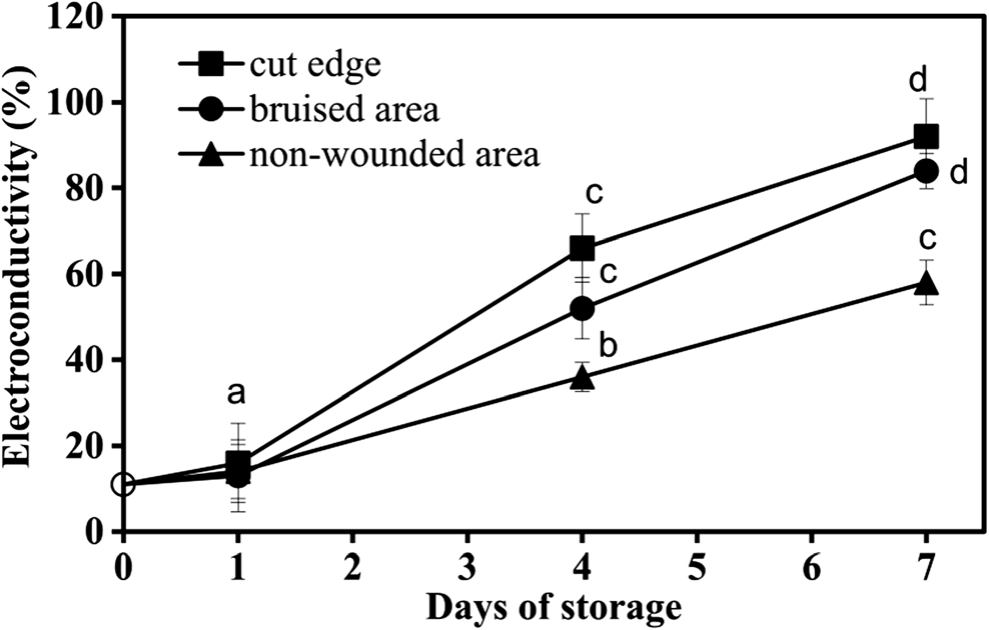
Electrolyte leakage of tissue discs from wounded (cut edge and bruised sites) and non-wounded areas in lettuce fresh-cuts, stored at 4 °C Presented values are means ± SEM _(*n*–1)_, (*n* = 9). At each time point, samples (15 leaf discs) were randomly taken from fresh-cuts from 3 boxes in three independent experiments with fresh-cuts prepared from 5 lettuce heads. Data indicated with same letters do not differ significantly from each other at *P* ≤ 0.05

The measurement of electrolyte leakage showed that the cell death in wounded and senescing sites was accompanied with an increase in electrolyte leakage (Fig. 8). It proceeded concomitantly with chlorophyll loss, browning and the augmentation of H_2_O_2_ and overall ROS production. On day 1 of storage, the electrolyte leakage in all samples was still similar to that of the initial samples (day 0). At day 4 and day 7, electrolyte leakage was lowest in the senescing areas (showing yellowing) and highest in the wounded areas (cut edge and bruised sites, showing browning) (Fig. 8). These data indicated that senescing and wounded cells of lettuce fresh-cuts were undergoing cell death, of which the electrolyte leakage is well recognised marker of compromised membrane integrity.

### Morphological occurrence of cell death

The dead cells were identified following the staining with Evans Blue and PI. Evans Blue positive dead cells were coloured in blue; PI positive nuclei in the dead cells emitted bright red fluorescence. After 1 day of storage, no dead cells were detected at the cut edge, bruised sites and non-wounded tissue (Figs. 5a, f and 6a, k). After 2 days of storage, cell death occurred in relatively low number of cells within the slightly browning area adjacent the wounded sites and this was accompanied with slightly elevated H_2_O_2_ level (Figs. 1a1, a2, a3).). Four days after cutting, most of the cells in the narrow zone close to the cut edge and at the sites of bruising were positive against Evans Blue and PI (Fig. 5b, c, g, h). In non-wounded areas of ‘non-bruised’ shreds, the cell death occurred throughout the entire senescing tissue (Fig. 6n, o) starting approximately a day later than in ‘bruised shreds’.

**Fig. 9:**
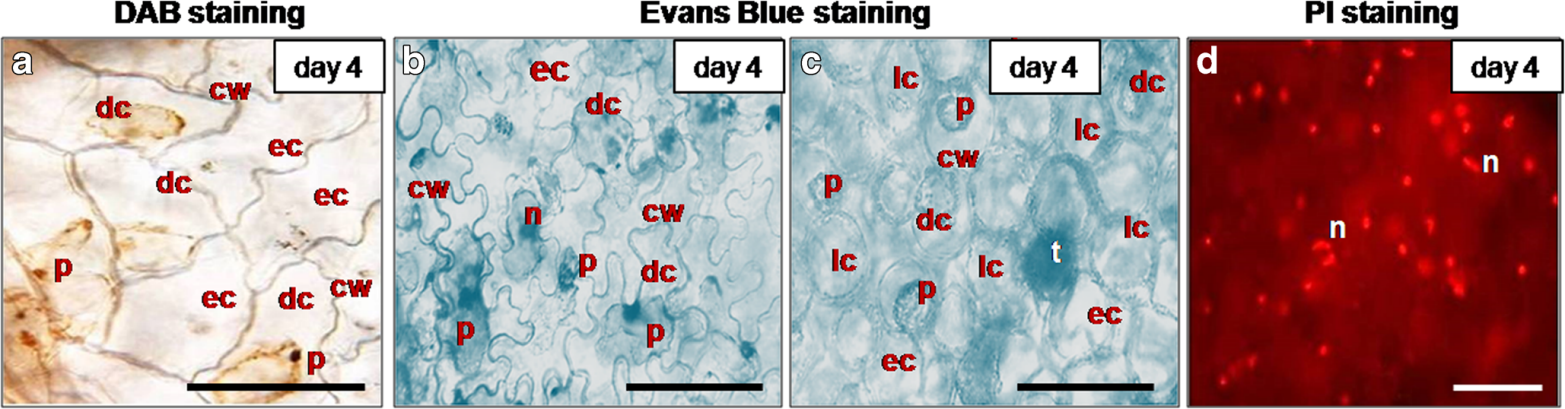
Expression of programmed cell death phenotype in wounded and senescing cells in lettuce fresh-cuts, stored at 4 °C **a** Dead epidermal cells (with accumulated H_2_O_2_) at the cut surface, DAB staining; note the brown coloured protoplasts. **b** Dead epidermal cells in senescing fresh-cut, Evans Blue staining. **c** Dead mesophyll cells in senescing fresh-cut (epidermis removed), Evans Blue staining. **d** PI stained condensed nuclei (bright red fluorescence) in dead cells in senescing sites. **a**–**c** Note the shrunken protoplast retracted from the cell wall and **b**, **d** Condensed nuclei. **a**–**c** Some of the cells appear empty, DAB and Evans Blue negative. Samples were taken on day 4 of storage. cw Cell wall, dc Death cell, ec Empty cell, lc Living cell, n Nucleus, p Protoplast, t Trichome. *Scale bars* = 100 μm

Propidium iodide-positive cells showed clear signs of condensed nuclei (Figs. 5c, h and 9d). Evans Blue staining revealed shrunken protoplasts separated from the cell walls both in the dead epidermal and in some of the dead mesophyll cells (Figs. 5g and 9b–c). Hydrogen peroxide remained within the shrunken protoplast (Fig. 9a). These features suggested necrotic type of PCD. Some of the cells appeared empty, Evans Blue negative (Fig. 9a–c). In the empty cells, H_2_O_2_ was not distinguishable by brown DAB deposits (Fig. 9a). The observations suggested that some of the cells might undergo vacuolar PCD, some might express necrotic and maybe others express mixed cell death phenotype.

### Role of trichomes in wound-induced senescence

On day 1 of storage epidermal and mesophyll tissue of non-bruised fresh-cuts consisted of living cells (Fig. 6a, k) and the trichomes contained only living cells (Fig. 6b). On day 2, it was noticed that before any visible signs of senescence, the cells in the upper part of single trichomes in ‘non-bruised’ shreds were apparently dead (Evans Blue positive) (Fig. 6l). At the base of same trichomes, the cells were Evans Blue negative. After 4 days of storage at senescing sites, the number of entirely dead trichomes increased (Fig. 6c, m) and dead (blue coloured) cells appeared also in the connecting epidermal tissue (Fig. 6m). The cell death progressed (Fig. 6n) and on day 7 most of the cells in epidermis and parenchyma were dead (Fig. 6o). Cell death advancement in trichomes and connecting tissue (Fig. 6l, m) was accompanied with H_2_O_2_ accumulation (brown DAB deposits) that further spread over the whole senescing area (Fig. 6g–j). These observations suggested that in fresh-cuts, the trichomes at sites distant from the primary wound sites may be the first to respond to and propagate the long distance wound signal.

## Discussion

Our earlier study suggested that wound stress-induced browning in lettuce fresh-cuts is associated with PCD symptoms (Iakimova and Woltering 2015). Here, we report further findings on morphological characterisation and signalling in wound-induced PCD. Most of the dead cells in the wounded and in non-injured (senescing) areas showed compacted nuclei and shrunken protoplast (retracted from cell wall). Some of the cells appeared empty suggesting that probably these are empty corpses remaining after autolysis of cellular content. Cell disappearance in the tissue undergoing highly advanced senescence substantiates the observations of Wagstaff et al. (2007) for vanishing cells in senescing detached leaves of postharvest lettuce heads. The observed cell death phenotypes resembling necrotic and vacuolar cell death classes suggested shared components of wound-induced cell death, senescence and HR PCD in lettuce. For example, phenotypic expression (e.g. protoplast shrinkage) of the dead cells together with activation of caspase 3-like protease and other elements of necrotic PCD are documented in pathogen challenged detached lettuce leaves and non-headed Chinese cabbage (Kiba et al. 2006, 2009; Li et al. 2006) whereas symptoms of vacuolar cell death are described in senescing lettuce leaves and in fresh-cuts subjected to high pressure stress (Wagstaff et al. 2007; Zhang et al. 2015). The changes in plasma membrane integrity and chlorophyll breakdown are markers of senescence and cell death in response to various stresses (Dhindsa et al. 1981; van Doorn and Woltering 2004; Lim et al. 2007; Song et al. 2012). Postharvest senescence in vegetable fresh-cuts suffering of storage-induced disorders, e.g. chilling injury, is also accompanied by membrane disruption and chlorophyll degradation (Artés et al. 2007; Hodges and Toivonen 2008; Saltveit 2002; Pedreschi and Lurie 2015; Pareek 2016). The observed in our experimental system increase in membrane permeability (electrolyte leakage) and chlorophyll loss indicate similarity between senescence and wound-induced cell death. The occurrence of dead cells of both phenotypes and the other detected physiological and biochemical markers of PCD in wounded and senescing tissue led to the assumption that part of the cells in lettuce fresh-cuts may undergo necrotic cell death and others may die in a manner of vacuolar cell death.

The biological role of the local wound-induced PCD in the vicinity of injured sites has been compared to the HR; both processes aimed at preventing the runaway spread of cell death (Cui et al. 2013; McCabe 2013). Our observations that cell death in lettuce fresh-cuts occurred quickly in the tissue close to the wound suggest that, in this system, PCD may function as a defence mechanism in order to rapidly seal-off the injury by physically separating the damaged from the healthy tissue with a layer of dead cells. In addition to the involvement of phenolics in the development of browning, it is thought that accumulation of polymerised phenolic compounds such as callose, suberin or lignin can play a role in building a physical barrier against propagating cell death response (Cui et al. 2013). Among the factors that are able to prevent the spreading of wound-induced death from the primary site of wounding throughout the rest of tissue is H_2_O_2_ which, apart from its role in PCD signalling (Levine et al. 1994; Jabs 1999; Neill et al. 2002), is involved also in pathways responsible for synthesis of antioxidant compounds that help the cells to cope with wound stress and can contribute to the strengthening of cell walls by participating in the cross-linking of its constituents (Cui et al. 2013; Tisi et al. 2008 and references therein).

Oxidative stress is involved in wound and other stress responses and is a substantial component of the PCD process (Jabs 1999; Sakamoto et al. 2005; Gill and Tuteja 2012). Hydrogen peroxide is recognised as localised mobile cell death factor in the HR PCD and in local wound signalling (Levine et al. 1994, 1996; León et al. 2001). We observed that the accumulation of H_2_O_2_ and other ROS corresponded to the advancement of browning and cell death at wounded sites. Obviously the oxygen species were produced in the wounded and in their neighbouring cells; additionally H_2_O_2_ may have diffused from the injured xylem vessels inside the area of cutting or bruising. This shows that ROS definitely contribute to the browning-associated confined wound response through mediating the cell death at the primary site of injury. Regarding the local cell death signalling, it is interesting to note that the presumed wound-induced primary signal molecules such as lysophospholipids (García et al. 2017) have previously been associated with the induction of cell death (necrotic PCD) in tomato cell cultures (Yakimova et al. 2006; Iakimova et al. 2013). The observed localised cell death at the wound site in fresh-cut lettuce may be triggered by these compounds. It addition, it is notable that a role of phosphatidylserine and its derivative lysophosphatidylserine is documented in animal cells undergoing apoptosis. These substances are involved in early apoptotic pathways and are also exposed on the outer surface of plasma membrane as a signal to phagocytes for recognising the apoptotic cells, thus promoting the phagocytosis—a process occurring in animal systems for removal of the remnants of apoptotic cells by macrophages (Denecker et al. 1999*;* Frasch and Bratton 2012*).*

The local browning, ROS production and cell death in wounded tissue occurred a day earlier than the first visible symptoms of senescence in non-wounded areas. In the later time points, ROS generation accompanied the course of senescence at the distant sites. Moreover, in comparison to the rapid increase of electrolyte leakage and chlorophyll loss at wounded sites, in non-injured tissue, these changes were delayed and expressed with lower severity. This suggested that ROS synthesis and the sequential senescence/cell death in non-wounded sites might be induced by a long-distance wound signal generated at the site of damage and transmitted toward the remote cells.

Potential players in long-distance stress communication, including wound signalling are jasmonic acid (JA), salicylic acid (SA), ethylene, NO, peptide messengers such as systemin, Ca^2+^-dependent pathways, MAPkinases, phospholipase A2, linoleic acid, octadecanoid pathways and electrical waves (López-Gálvez et al. 1996b; Ryan 2000; León et al. 2001; Campos-Vargas and Saltveit 2002; McCabe 2013). However, these factors might not operate collectively in the various stress situations and may function also as short-distance stress messengers. For example, Cui et al. (2013) assumed that wound-induced cell death is independent of SA, JA and ethylene but is related to abscisic acid (ABA). They found that ROS production is associated with ABA-stimulated local wound-induced cell death and showed that ABA additionally contributes to the spread of cell death away from the wound. The same authors also reported that the extent of dissemination of cell death is under control of the transcription factor BOTRYTIS SENSITIVE1/MYB108 which acts as a negative regulator of ABA production, hence preventing ABA-related long-distance wound signalling and limiting cell death to cells adjacent the wounds.

An intriguing question is how the senescence-inducing wound signalling was broadcasted to the unwounded parts of the lettuce fresh-cuts. We found that the first dead cells in non-injured areas appeared in trichomes. This was preceded by H_2_O_2_ accumulation in trichome cells. Further, H_2_O_2_ was intensively produced in the epidermal cells immediately connected to the respective trichomes and thereafter spread toward parenchyma. The cell death progression followed the same sequence. This suggested that trichomes might be the first structures distant from the wounded site that possibly can perceive a mobile signal generated in response to wound stress. In a model pathosystems, Wang et al. (2009) showed that trichomes can convey and incorporate external stress signals into intrinsic cellular pathways. These authors demonstrated that treatment of trichomes in tobacco leaves with the protein elicitor ParA1 from *Phytophthora parasitica* var. *nicotianae* activated HR PCD pathways occurring sequentially in trichomes and in the epidermal and mesophyll cells. An increase of H_2_O_2_ level was detected initially in the upper cell of trichome; next the H_2_O_2_ appeared in the lower trichome cells and thereafter in the connected epidermal and neighbouring mesophyll tissue. Our observations showed similar order of H_2_O_2_ production first in trichomes and later in the epidermal and mesophyll cells. This provides information suggesting that trichomes might participate in wound response of lettuce fresh-cuts by sensing and transmitting a long-distance wound signal which stimulates oxidative stress and additional events leading to cellular senescence (PCD). In support to our assumption is the discovery that *Arabidopsis thaliana* trichomes may function as mechanosensory system by sensing even slight folding, bruising, pressing or vibrations caused by insects touching the leaf surface or flying over it. Such disturbances were shown to induce changes in cytosolic Ca^2+^ and shift of pH toward alkaline state which in turn contributes to activation of pathways related to synthesis of plant defence toxins (Zhou et al. 2017). Another work suggested that tomato glandular trichomes can detect physical activity on the leaf surface and activate JA and H_2_O_2_ associated processes (Tooker et al. 2010). In velvet bean (*Mucuna pruriens*), insect-provoked mild mechanical stress has stimulated gene expression of a protein, containing domains belonging to papain family of cysteine proteases (Singh and Dhawan 2017). These proteases are known to be involved in diverse defence responses, HR and other PCD processes (Woltering 2010). The putative mobile signal that may potentiate senescence/cell death cascade at sites remote from the wounded lettuce tissue remains to be further elucidated.

## Conclusions

**Fig. 10:**
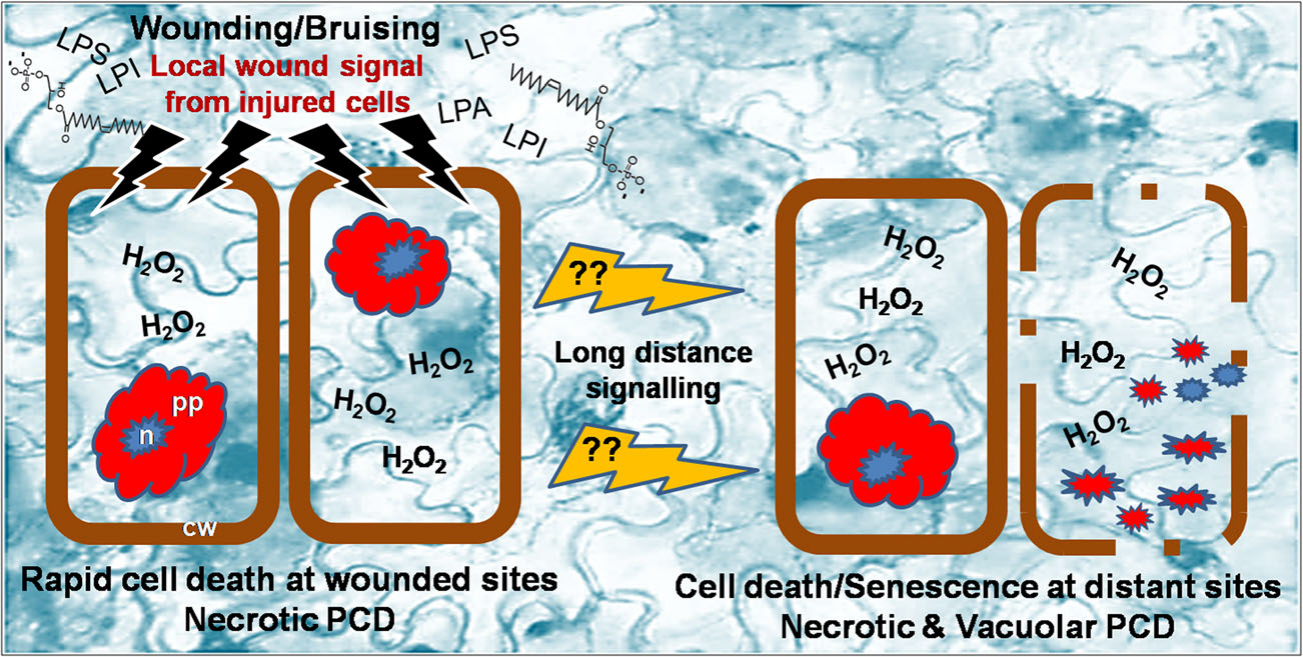
Schematic illustration of PCD involvement in the wound response in fresh-cut lettuce. Wounding (at the cut edge or bruised sites) involves the production of lysophospholipids (such as LPA, lysophosphatidic acid; LPS; lysophosphatidylserine; LPI, lysophosphatidylinositol) and causes rapid browning confined to the area surrounding the injured tissue. Browning is associated with massive cell death, H_2_O_2_ and general ROS accumulation, electrolyte leakage and chlorophyll loss. Dead cells mostly resemble necrotic PCD phenotype (shrunken protoplast). Dying cells generate signal molecules that travel over greater distances to cause ROS and cell death at distant sites; first in trichomes and subsequently in the connecting epidermal and mesophyll cells. In addition to necrotic PCD also vacuolar PCD (leaving empty cell corpses behind) and the complete disappearance of cells are observed. cw Cell wall, n Nucleus, pp. Protoplast

Our previous and current studies soundly indicate that PCD is an integral part of wound-associated browning disorder in lettuce fresh-cuts. Here, we present detailed characteristic of morphological, physiological and biochemical processes underlying the wound PCD response in this vegetable model. The morphological features of the dead cells (shrunken protoplasts and condensed nuclei, but also the appearance of empty corpses), together with H_2_O_2_ and overall ROS accumulation, compromised integrity of cellular membrane (electrolyte leakage) and presumable photosynthesis decline (chlorophyll loss) in both wounded and senescing sites pointed to cell death resembling a mixture of necrotic and vacuolar PCD types. The quick occurrence of cell death in vicinity of the wounds suggested that PCD may contribute to restricting the damage to the primary site of wounding by serving as a mechanism for building a physical barrier of dead cells between the injured and healthy tissue. The wound stress accelerated senescence in non-wounded tissue most probably through long-distance wound signalling. Trichomes in non-wounded sites were the first to show H_2_O_2_ accumulation and cell death followed by cell death in connecting epidermal tissue and consecutive senescence over larger area. This suggested a possible role of trichomes in mediating senescence/cell death at sites remote from the wounds (Fig. 10).

The findings add new information for the role of PCD in wound response in lettuce fresh-cuts and may open a path toward studies for controlling the deterioration in postharvest leafy vegetables through specifically targeting the PCD events.
